# Diffusion-weighted MR neurography for the assessment of brachial plexopathy in oncological practice

**DOI:** 10.1186/s40644-015-0041-5

**Published:** 2015-05-02

**Authors:** Adrian Andreou, Aslam Sohaib, David J Collins, Taro Takahara, Thomas C Kwee, Martin O Leach, David A MacVicar, Dow-Mu Koh

**Affiliations:** Department of Radiology, Royal Marsden, Sutton, Surrey UK; CR UK Clinical Magnetic Resonance Research Group, Institute of Cancer Research, Sutton, Surrey UK; Department of Radiology, University Medical Center, Utrecht, Netherlands

## Abstract

**Background:**

To evaluate diffusion-weighted MR neurography (DW-MRN) for visualizing the brachial plexus and for the assessment of brachial plexopathy.

**Methods:**

40 oncological patients with symptoms of brachial plexopathy underwent 1.5 T MRI using conventional MR sequences and unidirectional DW-MRN. The images were independently reviewed by two radiologists. Anatomic visualization of the brachial plexus was scored using a 5 point scale on conventional MR sequences and then combined with DW-MRN. A brachial plexus abnormality was also scored using a 5 point scale and inter-observer agreement determined by kappa statistics. Diagnostic accuracy for brachial plexopathy assessed by conventional MRI alone versus conventional MRI combined with DW-MRN was compared by ROC analysis using reference standards.

**Results:**

DW-MRN significantly improved visualization of the brachial plexus compared with conventional MRI alone (*P* < 0.001). When assessing brachial plexopathy, inter-observer agreement was moderate for conventional MRI (kappa = 0.48) but good for conventional MRI with DW-MRN (kappa = 0.62). DW-MRN combined with conventional MRI significantly improved diagnostic accuracy in one observer (*P* < 0.05) but was similar in the other observer.

**Conclusion:**

DW-MRN improved visualization of the brachial plexus. Combining DW-MRN with conventional MRI can improve inter-observer agreement and detection of brachial plexopathy in symptomatic oncological patients.

## Background

The brachial plexus is a network of nerves formed by the ventral branches of the spinal nerves C5 - T1 in the posterior triangle of the neck, which provides motor and sensory innervation to the upper extremity. The roots of the brachial plexus combine to form three trunks, which in turn divide to form anterior and posterior divisions. At the level of the lateral margin of the first rib, the divisions combine to form the three cords, which in the axilla give rise to the peripheral nerves of the upper limb.

Secondary tumours involving the brachial plexus are more common than primary neurogenic tumours. Brachial plexopathy from malignant disease most frequently arises from breast or lung carcinoma [1,2]. In oncological practice, the fundamental distinction is between tumour recurrence and non-malignant conditions such as fibrosis and inflammation, which may occur as a result of previous treatments such as chemotherapy, radiotherapy and surgery. Hence, accurate detection and depiction of brachial plexus pathology is important for effective management. In order to achieve this, the imaging technique should ideally allow clear structural visualization of the brachial plexus, from its origin at the cervical spinal cord to its terminal branches [3].

Conventional MRI has been applied to evaluate the brachial plexus. Most commonly, a combination of fat-suppressed T2-weighted (either frequency selective or short tau inversion recovery (STIR)) sequences and T1-weighted MR sequences are utilized [4,5]. However, the major disadvantage of these sequences is their inability to provide multi-planar or projection images that depict the entire length of the neural plexus. Furthermore, it may be difficult to differentiate the brachial plexus nerves from adjacent vascular structures [6]. As a consequence, subtle abnormalities can potentially be overlooked or missed.

Diffusion-weighted MR neurography (DW-MRN) has emerged as a promising method to visualize the peripheral nerves. The addition of a diffusion-weighted MR sequence to conventional MRI protocol may allow us to overcome some of the inherent disadvantages of conventional MRI [5,7]. DW-MRN uses the same principles as diffusion-weighted whole-body imaging with background body signal suppression [8]. DW-MRN allows the visualization of the spinal cord and peripheral nerves, because of their relatively lower apparent diffusion coefficient values, fibre orientation (diffusion anisotropy) and tissue organization. Furthermore, the use of a short-inversion-time inversion recovery prepulse for robust fat suppression over an extended field of view and heavy diffusion weighting (b 700 sec/mm^2^) ensure the suppression of unwanted signals from free fluid, fat, muscle and blood vessels. Image acquisition is performed in free breathing, resulting in images with high signal-to-noise for the same acquisition time compared with breath-hold and respiratory triggered acquisitions. This in turn, allows thinner slice acquisitions and multiple-slice excitations for multiplanar reformatting and display [7,8]. Although the imaging technique for DW-MRN has been described, its clinical utility for assessing patients with suspected brachial plexopathy in cancer has not been previously reported.

In our department, DW-MRN is used as part of the imaging protocol for the evaluation of brachial plexopathy in the patient with cancer. The purpose of this study was to evaluate DW-MRN for the visualization of the brachial plexus and the added value of DW-MRN to conventional MRI for the assessment of brachial plexopathy.

## Methods

The study was approved by the local research and ethics committee (Royal Marsden NHS Foundation Trust). Patient consent was waived because this was a retrospective review of prospectively acquired data.

### Study population

Forty consecutive patients (2 male, 38 female; mean age 57.0 years, range 38–64 years) with a known history of malignancy and symptoms of a brachial plexopathy were prospectively examined using conventional MRI and DW MRN of the brachial plexus. The inclusion criteria were: a) pathologically proven diagnosis of malignancy (29 breast, 2 lung, 9 others) and b) symptoms of brachial plexopathy (pain, weakness and/ or paresthesia). The exclusion criteria were any patient with contraindications for MRI or claustrophobia.

### Imaging technique

All patients were examined with a 1.5 tesla MR system (Siemens Avanto, Erlangen, Germany) using both a two-channel cervical spine and 16-channel torso phased array body coils. Conventional 2D MR sequences included T1-weighted images in the coronal, axial and parasagittal planes (TR = 410 ms, TE = 9.4 ms, FOV = 300 to 380 cm, Matrix size = 256 × 256, NEX = 16, 2.5 mm thickness) as well as STIR images in the coronal plane (TR = 3,000 ms, TE = 123 ms, T1 = 180 ms, FOV = 380 cm, Matrix size = 256 × 256, NEX = 16, 1.5 mm thickness). Axial T2-weighted were also obtained in the axial plane (TR = >5000 ms, TE = 80 ms, FOV = 300 to 380 cm, Matrix size = 256 × 256, NEX = 3, 2.5 cm thickness).

DW-MRN was performed using free-breathing single-shot echo-planar imaging with background body signal suppression, using a single b-value (700 sec/mm^2^). The choice of b-value is optimized for signal-to-noise when assessing white matter. Images were acquired from the level of C3 to T4 in all patients. Image acquisition was performed in the axial plane as direct coronal imaging tends to result in greater image artifacts because of the large field of view required and the difficulty in image shimming over this area. Unidirectional, antero-posterior motion probing gradients were applied as diffusion is relatively more impeded perpendicular to the long axis of the nerves, thus maximizing signal return. The following imaging parameters were applied: TR = 16,000 ms, TE = 69 ms, TI = 180 ms (STIR fat suppression), FOV = 300 cm, Matrix size = 150 × 150, NEX = 20, 2 mm isotropic voxel size, receiver bandwidth = 1450 Hz/pixel. A 40 mm coronal saturation slice was placed several cm anterior to the brachial plexus to suppress signal from soft tissues anterior to the brachial plexus as well as breathing and swallowing motion. The typical acquisition time was 5 minutes 40 seconds minutes.

The DW MRN data was post processed on a Siemen’s workstation (VB17 Leonardo). Multi-planar reformatted (axial, para-coronal and sagittal) and oblique (parallel to the long axis of the lower cervical spine) coronal maximum intensity projection (MIP) images were created and viewed using an inverted black-and-white grey scale. The reconstructed thickness of the MIP images was 40 mm.

### Image evaluation

Images from the symptomatic side were independently reviewed by two radiologists (**BLINDED**) with one year and eight years’ experience in body diffusion MRI, blinded to clinical and other radiological findings.

#### Anatomic visualization

**Figure 1:**
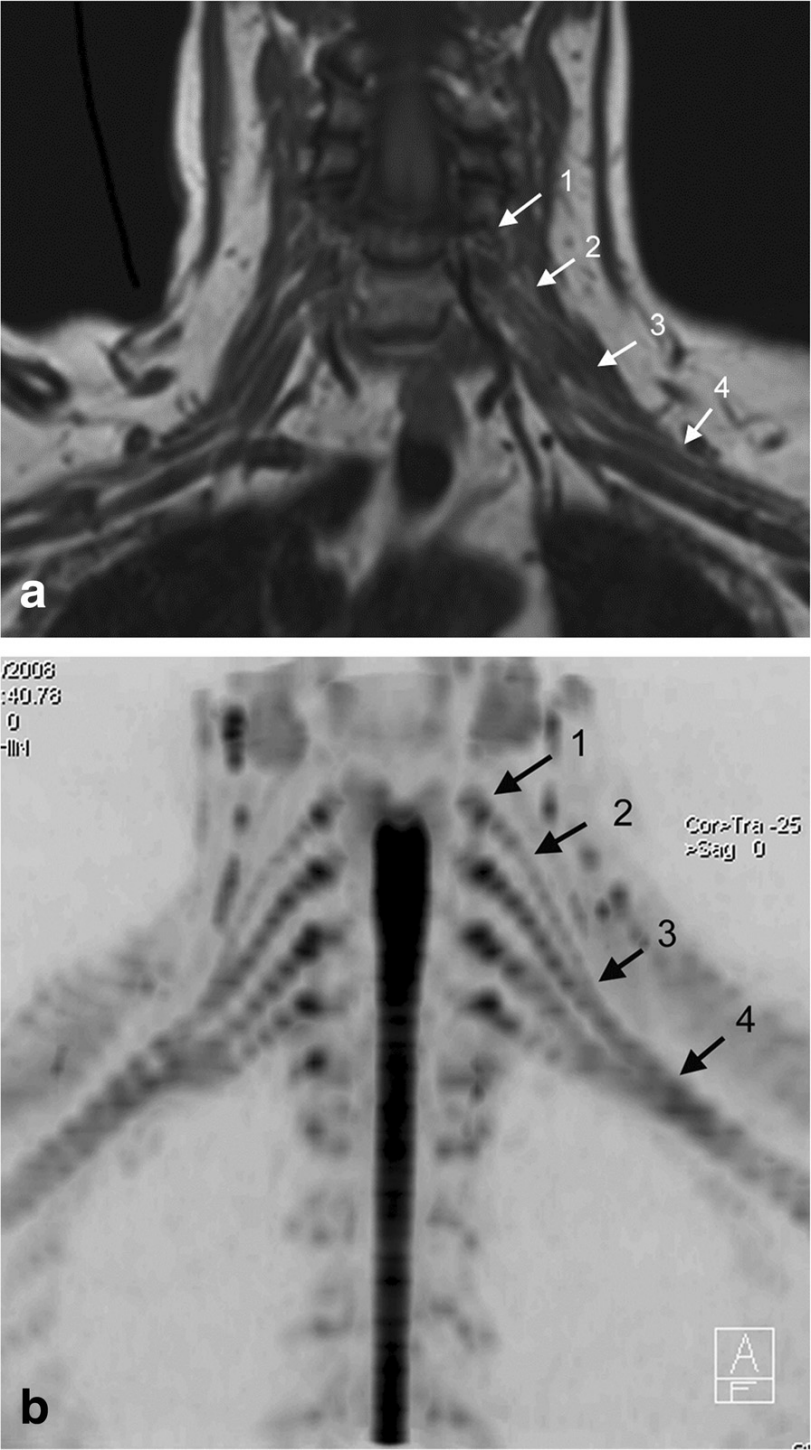
Anatomic evaluation of the brachial plexus on conventional imaging and DW-MRN. **(a)** Coronal T1-weighted image and **(b)** Inverted greyscale b = 700 s/mm^2^ coronal maximum intensity projection (MIP) from DW-MRN. The visibility of neural structures at the levels of the nerve ganglia, nerve roots, trunks, as well as divisions and cords (see arrows: 1 = ganglia, 2 = nerve roots, 3 = trunks, 4 = divisions and cords) were assessed visually.

The brachial plexus (C5 to T1) of the symptomatic side was systematically evaluated by assessing the: 1) ganglia, 2) roots, 3) trunks and 4) divisions and cords, on the conventional MR images and then again with the combination of conventional MR and DW-MRN (Figure 1). The ganglia and roots were scored as five separate levels (i.e. C5 to T1), whereas the trunks and the divisions and cords were assessed as three levels (upper, middle, lower), yielding a total of 16 regions assessed for each patient.

For each region, the anatomical depiction of the neural anatomy was scored on a 5 point scale (1 = not visible, 2 = just visible, 3 = patchy visibility and/or not contiguous with adjacent segments, 4 = mostly visible but not fully traceable into contiguous segments, 5 = clearly visible and traceable into contiguous segments).

#### Detection of brachial plexopathy

**Figure 2:**
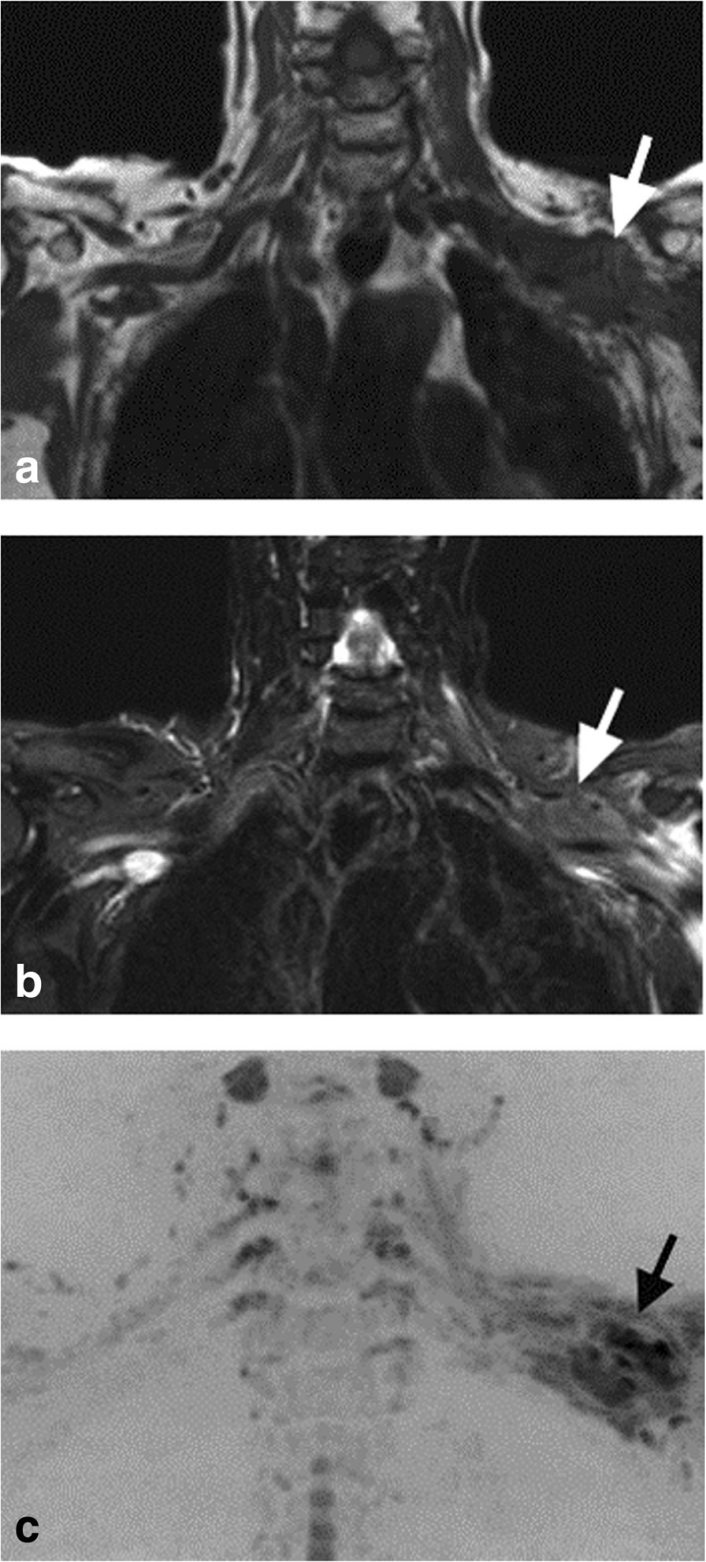
A 38 year-old women with history of left breast cancer presenting with left arm pain. **(a)** Coronal T1-weighted, **(b)** coronal STIR and **(c)** coronal inverted greyscale maximum intensity projection of the b = 700 s/mm^2^ images. Scans demonstrate irregular thickening at the level of the cord and divisions of the left brachial plexus, appearing of low T1-signal intensity, intermediate to high STIR signal intensity, and shows low signal intensity impeded diffusion (arrows). The disease in this case, was scored “5” on both conventional imaging and on the combination of conventional imaging and DW-MRN.

The presence of a brachial plexus abnormality at each of the anatomical region assessed as above was also scored on a 5 point scale (1 = normal, 2 = probably normal, 3 = indeterminate (smooth thickening), 4 = probably abnormal (focal nodular thickening or mass in contact with the nerves), 5 = definitely abnormal (diffuse nodular thickening or mass encircling/obscuring the nerves) (Figure 2). Non-visualized segments were scored as normal for the purpose of this analysis. The images were scored for conventional MRI alone and then the combination of conventional MRI and DW-MRN.

### Statistical analysis

A paired t-test was used to compare the mean scores for the anatomic visualization of the brachial plexus on a per region basis was compared for conventional MRI alone and the combination of conventional MRI and DW-MRN. Each region was regarded as independent for analysis.

The inter-observer agreement for the detection of a brachial plexopathy on a per region basis was determined by kappa statistics. The diagnostic accuracy for the presence of a brachial plexopathy using conventional MRI alone versus the combination of conventional MRI and DW-MRN was compared by receiver operating curve characteristics (ROC) analysis using the variance z-test. The reference standard was defined using clinical assessment (*n* = 40), follow up CT and MRI imaging *(n* = 40, mean duration = 16.4 months; range 12 to 44 months), 18 FDG PET-CT (*n* = 8) and biopsy (n = 3) as the reference standard. For all analyses, a *P*-value of <0.05 was deemed statistically significant.

## Results

In total 640 regions were assessed in our study population of 40 patients.

### Anatomic visualization

The combination of conventional MRI and DW-MRN significantly improved the anatomic visualization of the brachial plexus, compared to conventional MRI alone *(P* < 0.01). This improvement in visualization was seen at all levels of the brachial plexus assessed: neural ganglia (mean score 4.6 vs 2.6), roots (4.7 vs 2.2), trunks (4.3 vs 3.0), divisions and cords (4.2 vs 3.0), with the greatest improvement in visualization of the neural ganglia and nerve roots. The mean scores for the anatomic visualization are summarized in Table 1.

**Table 1:** **Mean scores for the anatomic visualization of the brachial plexus for conventional MRI alone and for the combination of conventional MRI and DW-MRN.**

	**Conventional**	**Conventional**	p-value*
	**MRI**	**MRI + DW-MRN**	
Ganglia	2.6	4.6	P < 0.001
Roots	2.2	4.7	P < 0.001
Trunks	3.0	4.3	P < 0.001
Divisions and Cords	3.0	4.2	P < 0.001

*p-values obtained by paired t-test.

The anatomic depiction of neural anatomy was scored on a 5 point scale (1 = not visible, 2 = just visible, 3 = patchy visibility and/or not contiguous with adjacent segments, 4 = mostly visible but not fully traceable into contiguous segments, 5 = clearly visible and traceable into contiguous segments).

### Detection of Brachial plexopathy

By the reference standard, 12 out of the 40 patients had a brachial plexus abnormality (10 malignant brachial plexopathy, 1 post-radiation neuritis, 1 neuroma). For the assessment of the presence of a brachial plexus abnormality, on a per region basis, the inter-observer agreement was moderate for conventional MRI alone (kappa = 0.48) but good for the combination of conventional MRI and DW-MRN (kappa = 0.62). These scores are summarized in Tables 2 and 3.

**Table 2:** **Inter-observer agreement for the assessment of the presence of a brachial plexopathy, on a per-region basis, using conventional MRI alone**

	**Observer 1**						**Kappa = 0.45**
							**95% CI = 0.33-0.56**
		**1**	**2**	**3**	**4**	**5**	
Observer 2	1	541	13	9	16	25	604 (94.45)
	2	0	3	3	0	0	6 (0.9%)
	3	3	0	0	0	3	6 (0.9%)
	4	3	0	0	3	0	6 (0.9%)
	5	0	0	0	0	18	18 (2.8%)
		547 (85.5%)	16 (2.5%)	12 (1.9%)	19 (3.0%)	46 (7.2%)	640

Scoring: 1 = normal, 2 = probably normal, 3 = indeterminate (smooth thickening), 4 = probably abnormal (focal nodular thickening or mass in contact with the nerves), 5 = definitely abnormal (diffuse nodular thickening or mass encircling/obscuring the nerves).

**Table 3:** **Inter-observer agreement for the assessment of the presence of a brachial plexopathy, on a per-region basis, using a combination of conventional MRI and DW-MRN**

	**Observer 1**						**Kappa = 0.59**
							**95% CI = 0.49-0.68**
		**1**	**2**	**3**	**4**	**5**	
Observer 2	1	545	7	8	6	25	591(92.3%)
	2	0	0	0	0	0	0 (0%)
	3	1	0	0	0	0	1 (0.2%)
	4	1	0	0	0	0	1 (0.2%)
	5	3	6	3	1	34	47.7 (7.3%)
		550 (85.9%)	13 (2.0%)	11 (1.7%)	7 (1.1%)	59 (9.2%)	640

Scoring: 1 = normal, 2 = probably normal, 3 = indeterminate (smooth thickening), 4 = probably abnormal (focal nodular thickening or mass in contact with the nerves), 5 = definitely abnormal (diffuse nodular thickening or mass encircling/obscuring the nerves).

**Figure 3:**
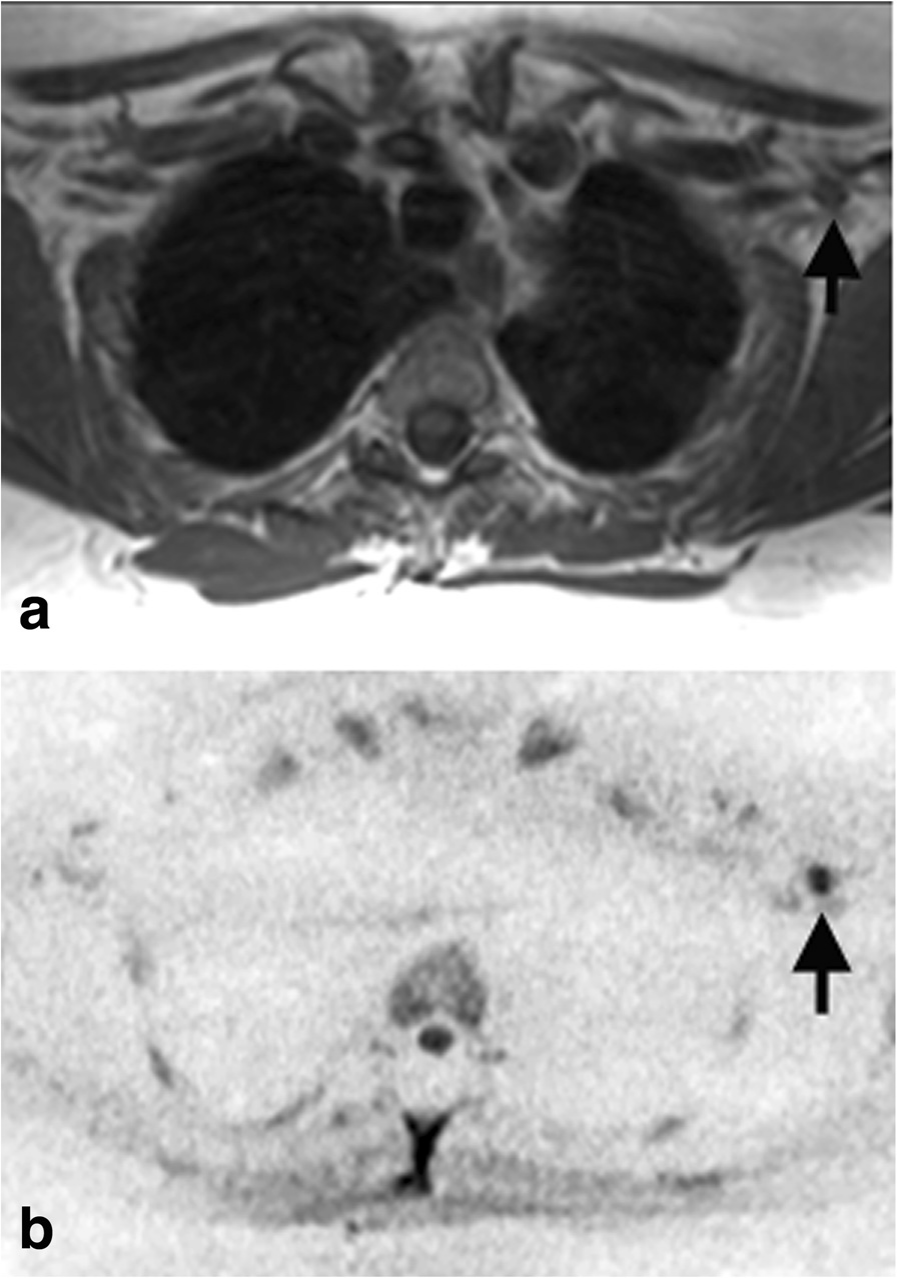
A 52 year-old women with breast cancer. **(a)** Axial T1-weighted and **(b)** paraaxial inverted greyscale maximum intensity projection (MIP) of b = 700 s/mm^2^ images. The DW-MRN images show a 4 mm nodular area of impeded diffusion (arrows), which is contiguous with the nerves, in keeping with a neuroma. There was no interval change at follow up imaging. The lesion was overlooked on T1-weighted imaging by one reader.

**Figure 4:**
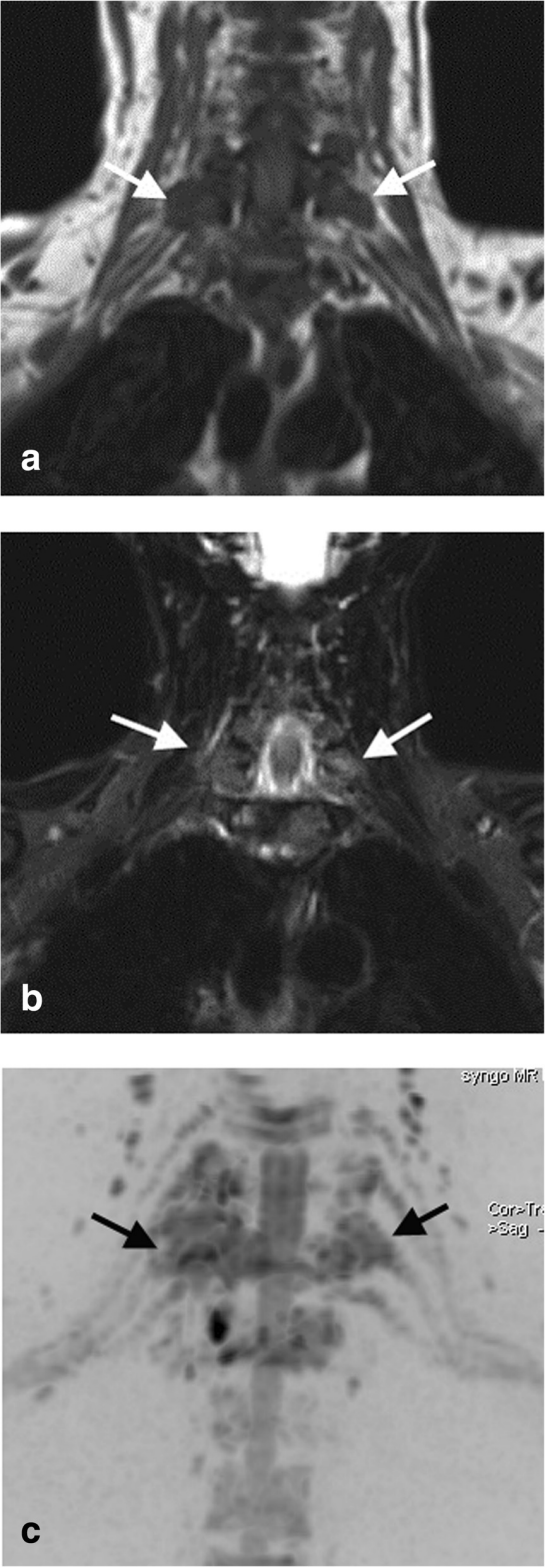
A48 year-old women with breast cancer with right arm symptoms of pain and numbness. **(a)** Coronal T1-weighted, **(b)** coronal STIR and **(c)** coronal inverted greyscale maximum intensity projection (MIP) of b = 700 s/mm^2^ images. On the MIP diffusion images, note abnormal impeded diffusion involving the right ganglia and nerve roots of C6 to C8, and also C7 on the left (black arrows). The disease was missed by one reader on the conventional imaging (white arrows).

On a per patient basis, by ROC analysis, DW-MRN combined with conventional MRI, significantly improved the diagnostic accuracy for detecting a brachial plexus abnormality in observer one (AZ = 1.00 vs 0.87, p < 0.05), but was similar for the second observer (AZ =0.96 vs 0.96), These results are summarized in Table 4. One reader missed the case of neuroma on conventional MRI, which was easily detected on DW-MRN (Figure 3). Another observer missed a case of malignant disease infiltrating the nerve roots (Figure 4). A case on DW-MRN was misinterpreted as disease in one reader because of image artefacts resulting in apparent thickening of the nerves.

**Table 4:** **Diagnostic accuracy on a per-patient basis by receiver operating curve characteristics analysis**

	**Conventional MRI**	**Conventional MRI + DW-MRN**	
	**Az values, (95% CI)**	**Az values, (95% CI)**	
Observer 1	0.87, (0.72 – 0.95)	1.00, (0.92 – 1.00)	p = 0.04
Observer 2	0.96, (0.84 – 0.99)	0.96, (0.84 – 0.99)	p = 0.96
	p = 0.11	p = 0.32	

Comparison of the diagnostic accuracies was performed using the variance z-test.

## Discussion

Conventional MRI sequences used to evaluate the brachial plexus typically include T1-weighted and fat-suppressed T2-weighted imaging. A major disadvantage of such conventional MRI techniques is their inability to produce MIP images, which aids visualization of the entire course of the neural plexus, due to the relatively unsuppressed signal from adjacent soft tissues [5]. Furthermore, it can be difficult to differentiate structures adjacent to the nerves, such as veins, as both these structures may have similar signal intensity on T1- and T2-weighted images [6].

The addition of DW-MRN to a conventional MRI protocol can help us overcome these limitations. DW-MRN improves the neural visualization by maximizing the contrast between the nerves and the signal suppressed background tissues. This enables MIP evaluation of the brachial plexus to be performed, allowing the nerves to be appraised over longer trajectories. The inclusion of DW-MRN to a standard MRI protocol does not incur significant time penalty, as the typical acquisition time is approximately 5 to 6 minutes. However, it has been reported even with the use of DW-MRN, visualization of the pre-ganglionic segments of the brachial plexus can still be suboptimal due to the small diameter of these nerves and cerebrospinal fluid flow fluctuations. Furthermore, depiction of the cervical nerves above C5 may also be unreliable because of their smaller diameters [5,9].

The technique of DW-MRN is adapted from body DW-MRI, which enhances the visualization of the spinal cord and peripheral nerves, due to their fibre orientation and tissue organization. In our study, unidirectional motion probing gradients were placed in the anterior-posterior direction, which is perpendicular to the trajectory of the brachial plexus nerves. This perpendicular positioning offers the highest signal return from the nerves, as diffusion is relatively more impeded perpendicular to the nerve [10]. In a study of DW-MRN of the sacral plexus, Takahara et al. demonstrated the superiority of DW MRN with unidirectional motion probing gradients, placed in the anterior-posterior direction, over DW-MRN using three or six directional motion probing gradients [11]. Motion probing gradients placed parallel to the peripheral nerves is least effective at visualizing them as diffusion is less impeded parallel to a nerve. As DW-MRI is also prone to image distortion, using multidirectional motion probing gradients may lead to different distortion directions, causing ineffective averaging of single-axis images to create the diffusion trace image, leading to image blurring and signal decrease of the diffusion trace image [11,12]. The additional saturation band reduces contributions from unsuppressed fat and other tissues, which leads to improved visualisation on the MIP images.

An accurate anatomic depiction of the brachial plexus is important for the detection and localization of disease. In this study we showed that the addition of DW-MRN to conventional MRI sequences, improved the anatomic visualization of the brachial plexus. The greatest improvement in neural visualization was in the proximal brachial plexus, including the ganglia and roots; a finding that was also observed by Takahara et al. [5]. This may be because the proximal segments have a more uniform trajectory, compared to the complex branching pattern of the more distal nerve fibres. Furthermore, the ganglia and roots are more spaced out and thus stand out more from the signal suppressed background tissues, compared to the more distal neural elements, which lie in closer proximity to other structures such as vessels, bone and lymph nodes.

The use of DW-MRN together with conventional MRI can improve the detection of brachial plexus abnormality. In our study, an improvement in diagnostic accuracy was seen in one out of the two observers, but was unchanged in the other. Nevertheless, it would appear that DW-MRI can aid the detection of disease along the brachial plexus. Furthermore, the inter-observer agreement for the presence of a brachial plexopathy on a per-region basis was improved when DW-MRN was combined with conventional imaging.

There are limitations to our current study. First, only oncological patients were evaluated. Further research should be performed to assess the diagnostic performance of DW-MRN in the detection of other conditions involving the brachial plexus, such as post-traumatic injury and neurogenic tumours. However, similar depiction of the brachial plexus using DW-MRN has been reported in studies involving small numbers of healthy volunteers and in non oncological patients by Yamashita et al. and Takahara et al. [5,7]. Second, histopathologic confirmation of a brachial plexus abnormality was only obtained in a small number of patients (3 out of 12), as biopsies in this region are not routinely performed, due to risk of injury to the large number of nerves and vessels in this area. Furthermore, disease at the apex of the axilla may not be readily accessible. However, in patients diagnosed with a brachial plexopathy who did not undergo biopsy, the diagnosis was using a combination of clinical assessment, other imaging such as CT and 18-FDG PET -CT and clinical follow up. Third, we utilized a standard MIP technique for viewing the DW-MRN images. It has been suggested that using the so called “soap-bubble” MIP technique [5] can improve neural visualization, but the technique was not available to us. Despite using the standard MIP technique, we were able to demonstrate advantages of DW-MRN. Last but not least, our conventional imaging protocol did not include STIR imaging in the sagittal plane. This is not part of the imaging protocol at our institution although it is widely employed. Instead, STIR imaging is performed in the coronal plane in our study. It is unclear whether this may have negatively biased our results for the assessment made using conventional imaging.

### Conclusion

DW-MRN improves the anatomic visualization of the brachial plexus. Using this technique, the conspicuity of the nerves was improved, which enabled MIP evaluation to be performed. Using DW-MRN in conjunction with conventional MRI improved inter-observer agreement for the detection of brachial plexus abnormality and can also improve the diagnostic accuracy in symptomatic oncological patients.
